# Systematic review of statistical methods for the identification of buildings and areas with high radon levels

**DOI:** 10.3389/fpubh.2024.1460295

**Published:** 2024-09-11

**Authors:** Joan F. Rey, Sara Antignani, Sebastian Baumann, Christian Di Carlo, Niccolò Loret, Claire Gréau, Valeria Gruber, Joëlle Goyette Pernot, Francesco Bochicchio

**Affiliations:** ^1^Western Switzerland Center for Indoor Air Quality and Radon (croqAIR), Transform Institute, School of Engineering and Architecture of Fribourg, HES-SO University of Applied Sciences and Arts Western Switzerland, Fribourg, Switzerland; ^2^Human-Oriented Built Environment Lab, School of Architecture, Civil and Environmental Engineering, Ecole Polytechnique Fédérale de Lausanne (EPFL), Lausanne, Switzerland; ^3^Italian National Institute of Health – National Center for Radiation Protection and Computational Physics, Rome, Italy; ^4^Austrian Agency for Health and Food Safety, Department of Radon and Radioecology, Linz, Austria; ^5^Institut de Radioprotection et de Sûreté Nucléaire, Bureau d'Etude et d'expertise du Radon, IRSN, PSE-ENV, SERPEN, BERAD, Fontenay-aux-Roses, France

**Keywords:** radon prone areas and building, public health, statistic, geostatistics, machine learning

## Abstract

Radon is a natural and radioactive noble gas, which may accumulate indoors and cause lung cancers after long term-exposure. Being a decay product of Uranium 238, it originates from the ground and is spatially variable. Many environmental (i.e., geology, tectonic, soils) and architectural factors (i.e., building age, floor) influence its presence indoors, which make it difficult to predict. However, different methods have been developed and applied to identify radon prone areas and buildings. This paper presents the results of a systematic literature review of suitable statistical methods willing to identify buildings and areas where high indoor radon concentrations might be found. The application of these methods is particularly useful to improve the knowledge of the factors most likely to be connected to high radon concentrations. These types of methods are not so commonly used, since generally statistical methods that study factors predictive of radon concentration are focused on the average concentration and aim to identify factors that influence the average radon level. In this paper, an attempt has been made to classify the methods found, to make their description clearer. Four main classes of methods have been identified: descriptive methods, regression methods, geostatistical methods, and machine learning methods. For each presented method, advantages and disadvantages are presented while some applications examples are given. The ultimate purpose of this overview is to provide researchers with a synthesis paper to optimize the selection of the method to identify radon prone areas and buildings.

## 1 Introduction

Radon is a naturally occurring radioactive gas that might accumulate indoors and pose a health issue. It is formed from the decay of uranium found in soil, rock, and water (1, 2). Radon can enter buildings through cracks in foundations, gaps around pipes, and other openings, where it can accumulate to potentially harmful levels (2, 3). Prolonged exposure to elevated radon concentrations increases the risk of developing lung cancer, making it a leading cause of lung cancer (2, 4) along with smoking. Furthermore, high radon exposure affects individuals of all ages and backgrounds, with particularly heightened risks for smokers due to the synergistic effect of radon and smoking fumes (5, 6). For these reasons, it became a public health issue, handled by the WHO since the beginning of the 1980’s (2). Radon management, regulations and their implementation vary significantly from one country to another, but the common goal is to reduce the average level of radon to which the population is exposed and thus reduce the risk. Each nation adopts its approach to address the challenges posed by radon exposure based on factors such as geographical location, geological composition, and existing infrastructure. European Basic Safety Standards, Euratom Directive 59/2013, requires that Member States identify areas with elevated levels of indoor radon concentration. According to the optimization principle in radiation protection, areas identified as radon-prone and buildings with high radon concentrations are priority targets for intervention to reduce radon-related risks.

Measuring radon levels typically involves deploying dosimeters in buildings, dwellings, schools, and workplaces to assess the concentration of radon gas over a specified period. However, conducting radon measurements in every building is barely impossible due to logistical challenges and resource constraints. The sheer number of buildings, coupled with varying access permissions and the need for prolonged monitoring periods, as all the measurement protocols require, makes comprehensive testing unfeasible. Therefore, it is crucial to explore alternative approaches to identify radon-prone areas and buildings efficiently.

Indoor radon levels can be influenced by various factors, including geological, climatical, building and occupancy characteristics (7). The presence of uranium-rich soil and rocks beneath or surrounding a building can significantly influence indoor radon levels (8–10). The lithology of the region, including the type of rocks and soil composition, plays a crucial role in determining the potential for radon generation (8). Additionally, climatic factors such as temperature, humidity, atmospheric pressure and precipitation can affect radon transport and accumulation within the soil and surrounding environment (7, 11–14). Furthermore, tectonic activity, such as faults and fractures in the Earth’s crust, can create pathways for radon gas to migrate from deep geological layers to the surface and into buildings (15–17). Therefore, a comprehensive understanding of the geological, climatic, and tectonic characteristics of an area is essential for assessing and mitigating indoor radon levels effectively. Moreover, various building factors can influence the presence of radon and its concentration levels indoors. Construction materials and building design play a significant role in determining radon infiltration (18–21). For instance, the presence of cracks in the foundation or walls can provide pathways for radon gas to enter a building from the surrounding soil (3). Additionally, the type of flooring, the age and the type of building may all influence indoor radon concentrations (22, 23). The ventilation system of a building also plays a crucial role in radon mitigation, as proper ventilation can help dilute radon concentrations and more generally promotes a better indoor air quality (18, 24, 25). Moreover, occupancy patterns, such as the number of occupants, the duration of time spent indoors and their activities, can influence radon levels and indoor air quality by affecting indoor air circulation and mixing (7, 22, 26).

A comprehensive understanding of the factors influencing indoor radon levels is essential for guiding well-defined and sustainable public health policies to mitigate the health risks associated with elevated indoor radon levels. By investigating and analyzing these different influences on indoor radon levels, and their inter-relationship, it may be possible to predict indoor radon levels, or at least, the probability of exceeding specific radon levels. These approaches include basic statistical analysis, regressive statistical analysis, geostatistical methods and machine learning (ML) methods. In today’s landscape, the abundance of available methods poses a challenge for researchers in selecting the most appropriate method relative to its own context of application. This paper thus aims to delve into main methods used for investigating indoor radon concentrations, with a special focus on high levels, and to assess their respective applications. More specifically, this paper seeks to present a systematic review of statistical methods to identify radon prone areas and buildings as a priority target of intervention to reduce radon related risk, aiming to comprehensively evaluate effectiveness and suitability of the different methods across different contexts and scenarios.

## 2 Methodology

### 2.1 Document selection

The literature searches for methods aiming at (1) identifying areas with high radon levels and at (2) identifying buildings with high radon levels that have been performed separately. However, for both purposes, a systematic review of the published studies has been performed using the PRISMA methodology (27). The latter methodology is commonly used by researchers to perform systematic literature review and meta-analysis (27). Among the databases available, we used the following online databases to search for documentation: Web of Science, Scopus and PubMed.

The defined research strategy aimed to collect all the documents, regardless of the document type, the year of publication, the language, and its availability (i.e., open source). All documents have been collected up to the 10th of May 2024. The keywords used for the searches are reported below in Table 1 for the identification of radon prone areas and in Table 2 for the identification radon prone buildings. The wildcard “*” was used to encompass different words that are variations of the same term. The references in the retrieved articles were also assessed as potentially relevant.

**Table 1:** Research criteria to select documents for identifying radon prone areas.

Criteria	Where	Keywords
I	Title OR Abstract	“radon” | “(222)Rn” | “222Rn” | “Rn-222”
II	Title OR Abstract	“radon prone area*” | “priority area*” | “high radon level*” | “elevated radon level*” | “high radon concentration*” | “high radon exposure*” | “high radon potential” | “high background” | “radon affected area*”
III	Title OR Abstract	“method*” | “approach*” | “mapping”
IV	Title OR Abstract	NOT “transform*”

Each document selected contained one keyword, at least, in each criterion keywords list. “Or” is implemented with its logical sign: “|.” The wildcard “*” was used to encompass different words that are variations of the same term. The criteria I-IV were combined using the “And” Boolean operator.|.” The wildcard “*” was used to encompass different words that are variations of the same term. The criteria I-VI were combined using the “And” Boolean operator.|.” The wildcard “*” was used to encompass different words that are variations of the same term. The criteria I-IV were combined using the “And” Boolean operator.|.” The wildcard “*” was used to encompass different words that are variations of the same term. The criteria I-IV were combined using the “And” Boolean operator.

**Table 2:** Research criteria to select documents for identifying radon prone buildings.

Criteria	Where	Keywords
I	Title OR Abstract	“radon” | “(222)Rn” | “222Rn” | “Rn-222”
II	Title OR Abstract	“building*” | “dwelling*” | “hous*” | “school*” | “workplace*” | “indoor*”
III	Title OR Abstract	“factor*” | “characteristic*” | “feature*” | “parameter*”
IV	Title OR Abstract	“identif*” | “detect*” | “predict*” | “forecast*” | “affect*” | “influenc*” | “impact*”
V	Title OR Abstract	“high*” | “elevated” | “exceed*” | “above” | “quantile*”
VI	Title OR Abstract	“approach*” | “method*”
VII	Title OR Abstract	“logistic regression” | “quantile regression” | “random forest” | “machine learning”

Each document selected contained one keyword, at least, in each criterion keywords list. “Or” is implemented with its logical sign: “|.” The wildcard “*” was used to encompass different words that are variations of the same term. The criteria I-IV were combined using the “And” Boolean operator.|.” The wildcard “*” was used to encompass different words that are variations of the same term. The criteria I-VI were combined using the “And” Boolean operator.|.” The wildcard “*” was used to encompass different words that are variations of the same term. The criteria I-IV were combined using the “And” Boolean operator.|.” The wildcard “*” was used to encompass different words that are variations of the same term. The criteria I-VI were combined using the “And” Boolean operator.

### 2.2 Data extraction and evaluation of the papers

**Figure 1:**
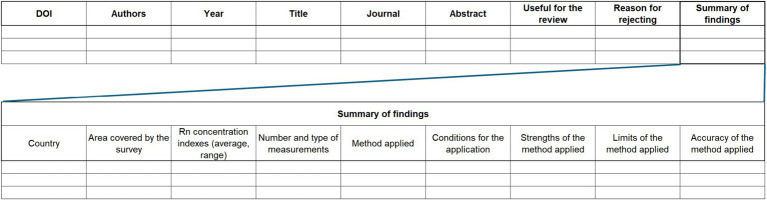
Standardized form used to evaluate the selected documents.

Selected documents were evaluated and information about each one was extracted using a standardized datasheet. The latter was used to collect: (1) main information of the paper evaluated; (2) main characteristics of the datasets to which the method has been applied (e.g., number and type of measurements); (3) main information of the method used (e.g., pro and cons, accuracy, conditions for applicability). Based on this information, the paper was either rejected or retained for inclusion in the review. The standardized form used in this study is presented in Figure 1.

### 2.3 Classification of the results

The systematic literature review resulted in a large and dense dataset, both in terms of identified methods and their application scope. Although the literature search has been conducted separately on statistical methods for identifying buildings and areas with elevated radon levels, we present the methods without distinguishing between their areas of application, as many methods serve both purposes. Aiming to ease the presentation of the results, we classified the different methods according to their type. The *Application* section of each type of method lists the work in which the method has been used, highlighting their application scope, which is often guided by the type of input data. The proposed classification is not a clear-cut classification, since some methods described in different classes may have features in common. Four broad categories were identified: (1) descriptive statistical methods; (2) traditional regression (quantile and logistic) methods; (3) geostatistical methods; (4) machine learning methods.

The methods selected for this review will be described in sections dedicated to the four distinct categories, with relevant literature cited as examples of their application. All the methods can generally be used for mapping purposes, that is to characterize a territory in terms of its radon potential thus identifying areas with high radon concentrations. With this aim, these methods might be applied to different types of data, such as indoor radon data or soil radon concentration measurement data. Alternatively, the same methods can be used to identify factors that predict high concentrations in buildings; in this case, the data of interest are typically indoor radon data.

### 2.4 Evaluation of the methods performances

Working with predictive models (or algorithms) leads to the need to evaluate the performance of a model, that is its ability to make accurate predictions on unseen data.

Several metrics exist to evaluate the performance of a model, and the choice depends on the type of model, whether it is either a regression model (continuous output) or a classification model (nominal or binary output). To test how well a predictive model generalizes, the data set is usually split into training and test data, where the test data is only used for performance evaluation and should be independent from the training set. Alternatively leave-one-out cross validation (k-folding) can be used to test the performance.

In *k-folding* the dataset is subdivided into *k* different folds (dataset portions) which are in turn extracted to test the model built on the remaining (*k-1*) dataset. Since the model is trained and validated multiple times on different subsets of the data, that “crossed” between training and validation roles, k-fold cross-validation helps to reduce the risk of overfitting, which occurs when a model performs well on the training data but poorly on unseen data.

**Figure 2:**
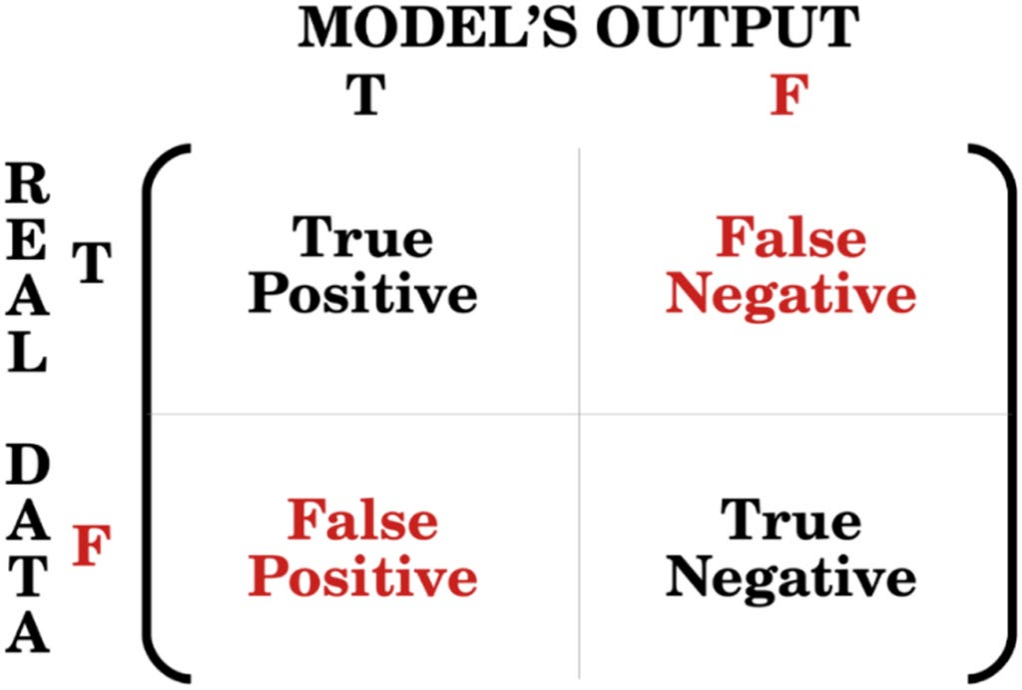
Relationship between real data and model’s output within a confusion matrix.

A widely used tool for measuring the performance of a classification model (e.g., a logistic model with a binary outcome) is the confusion matrix (Figure 2), from which various performance metrics, such as accuracy and AUC-ROC curves. Are calculated.

Accuracy is a measure of how well the models’ predictions fit real data, based on the number of correct (true) predictions out of all predictions made. The AUC (Area Under the Curve) measures the area underneath the Receiver Operating Characteristic (ROC) curve, that is the plot between the true positive rate (also known as sensitivity) and the false positive rate (also known as 1-specificity).

Both the accuracy and the AUC-ROC metrics range from 0 to 1, where a higher value indicates better model performance. A value of 0.5 suggests that the model’s predictions are not meaningful or useful for distinguishing between classes in the dataset, that is, the model has no discriminative power (equivalent to random guessing). Thus, the 0.5 value could be used as a benchmark for comparison.

Among the most used metrics to evaluate the performance of a regression model are MAE (Mean Absolute Error), that measures the average absolute differences between predicted and actual values, RMSE (Root Mean Squared Error), that is based on the differences between predicted and actual values, and the *R*-squared, which measures the proportion of the variance explained by the model. A better model’s performance is associated with higher *R*-squared values and lower MAE or RMSE values. When the RMSE decreases or the *R*-squared increases, the model’s performance improves.

## 3 Results

### 3.1 Document selection

#### 3.1.1 Radon prone areas

**Figure 3:**
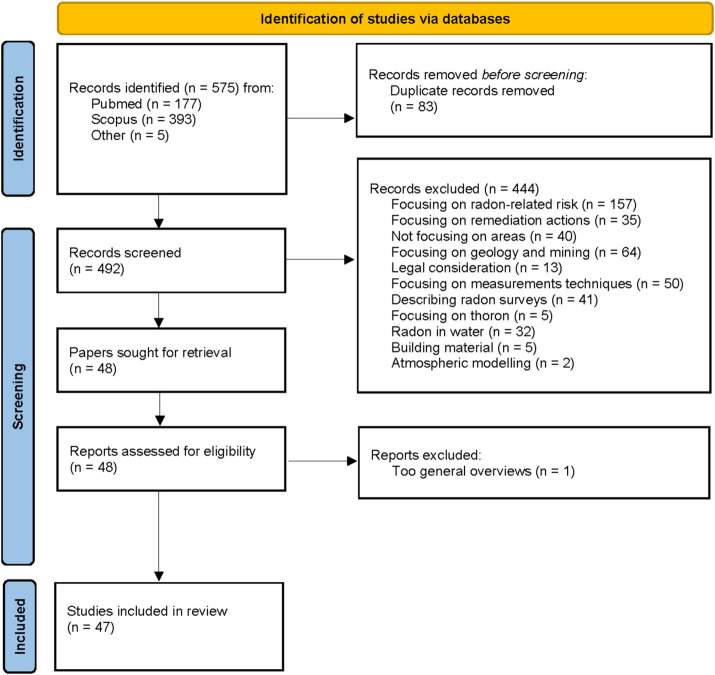
Flowchart of the included papers to review the methods to identify areas with high radon levels. Based on PRISMA 2020 flow diagram template (27).

The process of documents selection and exclusion is depicted in Figure 3. Overall, 575 records were found in two databases (PubMed, Scopus) and duplicate records were removed. A total of five publications were added due to personal communication which sums up to a total of 492 selected publications. All identified papers were in English.

After screening titles and the abstracts of these papers, 445 papers were discarded for different reasons. The major reason for neglecting a publication was that the focus was brought on general radon risk, geology and mining or measurement techniques. Afterwards, another six publications were discarded due to a too broad and general overview. At the end, 47 publications remained and were further examined and analyzed for the identification of areas with high radon levels.

#### 3.1.2 Radon prone buildings

**Figure 4:**
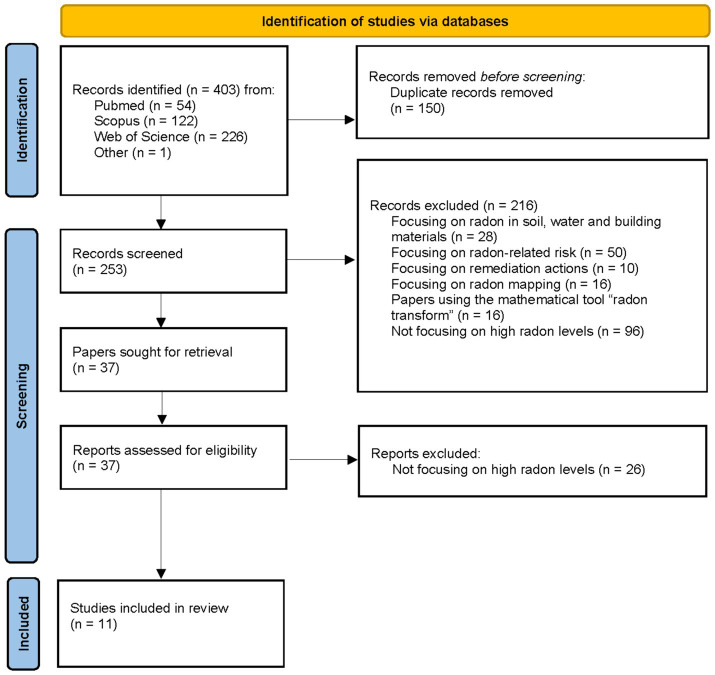
Flowchart of the included papers to review the methods to identify buildings with high radon levels. Based on PRISMA 2020 flow diagram template (27).

A total of 252 papers were identified after removing the duplicates from the 403 records found. All identified papers were in English. The process of study selection and exclusion is shown in Figure 4.

After the screening of the titles and the abstracts of these papers, 216 papers were discarded since they did not deal with the issue of interest for the present review (i.e., mostly papers describing methods able to find explanatory variables of the average indoor radon levels within buildings). One additional paper found among the references of the retrieved articles was also preselected. Overall, 37 full text articles were further examined. Of these, 11 papers were deemed of interest as they specifically address methods aimed at identifying buildings with high radon concentrations.

### 3.2 Focusing on predicting high radon levels

Different approaches to focus on the prediction of high radon levels exist. In our work we are mainly interested in methods that predict areas and buildings with high radon levels. Therefore, predictive models can be powerful tools and with a solid database and a profound training and test data split it is possible to create accurate models that generalize. In general, predictive models are not designed to predict certain values or value ranges like very small or very high values. But modifying the target value it is possible to focus on special value ranges, for example to focus on high radon values. Different techniques to increase the impact of high values exist.

One strategy to focus on high radon levels is to keep the actual radon concentration as target value without transformation or modification. Indoor radon concentrations typically approximately follow a log-normal distribution (28). The lognormality of the target variable in a predictive model is an interesting feature, moreover when focusing on high radon levels, where the few but not often occurring high radon levels might be the most interesting ones. In a regression task when evaluating the model performance, the error of prediction and target value are calculated. The error of predictions is commonly a measure of distance, as the mean squared error or mean absolute error. Observations with higher deviations of prediction and target value contribute more to the error than observations with low deviations. Transforming the target value changes how observations with high indoor radon concentrations contribute to the prediction error. If the actual log-normal distributed indoor radon concentrations without a transformation or aggregation are used as target variable, higher indoor radon concentrations can contribute more to the prediction error than low concentrations, because they have a higher potential for deviation. By log-transforming the indoor radon concentrations this effect vanishes and the potential of contributing to the prediction error becomes balanced, and the focus of prediction is shifted from observations with high indoor radon concentration to average values. Therefore, predicting the log-transformed target value of a log-normal distribution increases model performance and because great deviations from the average values are less penalized.

The aggregation of indoor radon concentrations by using the geometric mean or other central aggregations has a similar effect on the predictions. By transforming or aggregating the indoor radon concentrations higher indoor radon concentrations lose impact, but model performance will increase. Using the untransformed log-normal distributed indoor radon concentrations is an example of increasing the impact of high values by penalizing them during training. Similar results can be achieved when weighing the samples according to their target value, where higher values get higher weights and have a greater impact on the performance evaluation.

Duplicating samples with high values can have a similar effect. Doing so no new information is added to the data set but during training the model more often sees these samples and therefore tries best to predict these high values. As indoor radon concentrations typically show a log-normal distribution, high values are underrepresented and by duplicating these high values the data set gets more balanced.

By transforming the regression into a classification task, the performance evaluation shifts from a distance metric yes/no decision for each class, where the exact value of the target value is not important anymore but the overall class. For example, indoor radon concentrations could be divided in two classes, lower and greater equal to 300 Bq/m^3^. This could lead to more robust predictions for each class and therefore also for high radon levels,

Another strategy is to focus on the prediction on high radon levels is to adopt the loss function, as will be discussed in the quantile regression section and machine learning chapter, where machine learning methods can be transformed to a quantile regression task by using the tilted absolute value loss function for neural networks or by using quantile regression forests, which is an adoption of random forests.

### 3.3 Statistical methods to identify areas and buildings

Four broad classes of methods have been identified: (1) descriptive statistics methods; (2) regression methods; (3) geostatistical methods; (4) and machine learning methods. These classes of methods are presented below, with the aim of highlighting their respective strengths, limitations, and performance, if reported.

#### 3.3.1 Descriptive statistics methods

Descriptive statistical methods aim to analyze, summarize, and explore data without the willingness to predict. These methods are mandatory to explore data, which includes the calculation of basic aggregates as means or ranges, or the visualization through different kind of plots, i.e., correlation analysis, ANOVA, or outlier detection. Descriptive statistical analysis is therefore the basis of any further investigation or predictive method, while it also produces its own findings. For example, these methods are used to produce maps describing a territory based on measurements of a certain variable. Two main descriptive statistical methods were applied to indoor radon dataset, presented in Table 3. These methods are correlation analysis, and statistics by geographic grouping.

**Table 3:** Selected descriptive statistics methods and their respective descriptions.

Method	Description
Correlation analysis	Correlation is a statistical measure which provides information about the relationship between two variables. The strength of the linear correlation is determined by the correlation coefficient ranging from −1 to +1. Correlation analysis does not imply causation but rather can be used to determine the strength and direction of the correlation between two variables.
Statistics by geographic grouping	When data are georeferenced and numerous, summary statistics (e.g., arithmetic mean, standard deviation, ...) of the variable of interest can be produced by geographic grouping such as grids-square or geological/administrative units.

Correlation analysis has been applied in different context to assess the statistical link (e.g., Pearson’s correlation coefficient) between indoor radon and geological data (29–32), uranium content data (30), gamma ray survey (30, 33) or radon-222 exhalation rate measurements (34). Indeed, indoor radon measurements are generally used to confirm a mapping of a high radon area when the map was previously created with another dataset than indoor radon concentration (e.g., geology, field measurement) (30, 34, 35).

A common approach to delineate radon prone areas is based on the calculation of descriptive statistics (from a dataset of radon concentration measurements) typically for each of the administrative units in a territory (36–39). Miles and Appleton (40) applied some statistics by geographic grouping by combining grid square and geological mapping methods. The resulting maps were more accurate: within each geological combination with more than 100 radon measurements, the variation of radon potential was mapped using a 1 km^2^ of the national grid. Radon potential was attributed to each grid square based, at least, on the nearest 30 house radon measurement results to that square. Grouping indoor radon measurements with geology on 1 km^2^ of the national grid squares allowed to identify new radon affected areas that remained unidentified so far, especially in areas where radon measurements were rare. However, high indoor radon levels measured within a single square with low measurement density may overestimate the potential.

#### 3.3.2 Regression methods

Regression is a common statistical method for studying relationships between variables. In general, a response variable *Y* is a function of a set of *n* observed predictor variables *X_1_,.,X_n_*, also defined as covariates, such that *Y = f(X_1_,.., X_n_)*. With regression methods the only information that is obtained about the relationship between y and the covariates *X* is how the *mean* of the response variable *Y* varies as each *X_i_* varies; in other words, the function is defined for the expected value of *Y* conditional on the covariates:


(1) $$E\left(Y|X_{1},,\ldots ,,X_{n}|\right)=\beta_{0}+\beta_{1}X_{1}+\ldots +\beta_{n}X_{n}$$

With the coefficients $\beta_{i}$ describing the impact of each covariate on the average level of the response variable. Focusing exclusively on variations in the average may under or overestimate or fail to detect the real impact some variables may have on a response variable. If, therefore, we apply a classical regression model that predicts effects of several factors (predictors) on the mean value of radon concentration, the results obtained may not provide a complete picture of the relationships between those variables, especially if we are interested in the impact that certain factors have on high radon concentration values (right tail of the radon concentration distribution).

##### 3.3.2.1 Quantile regression

It is possible to fit regression curves to other parts of the distribution of the response variable: the quantile regression, as introduced by Koenker and Bassett (41), is a method for estimating functional relations between variables for all portions of a probability distribution, thus implying the possibility that there is not a single rate of change describing the relationship between a response variable and predictor variables. Quantile regression extend the classical regression methods aiming to estimate the relationship between quantiles of the conditional distribution of the response variable Y and a set of observed covariates X_1_,..,X_n_ (Equation 2):


(2) $$Q_{Y}\left(\tau |X_{1},,\ldots ,,X_{n}|\right)=\beta_{\tau 0}+\beta_{\tau 1}X_{1}+\ldots +\beta_{\tau n}X_{n}$$

**Figure 5:**
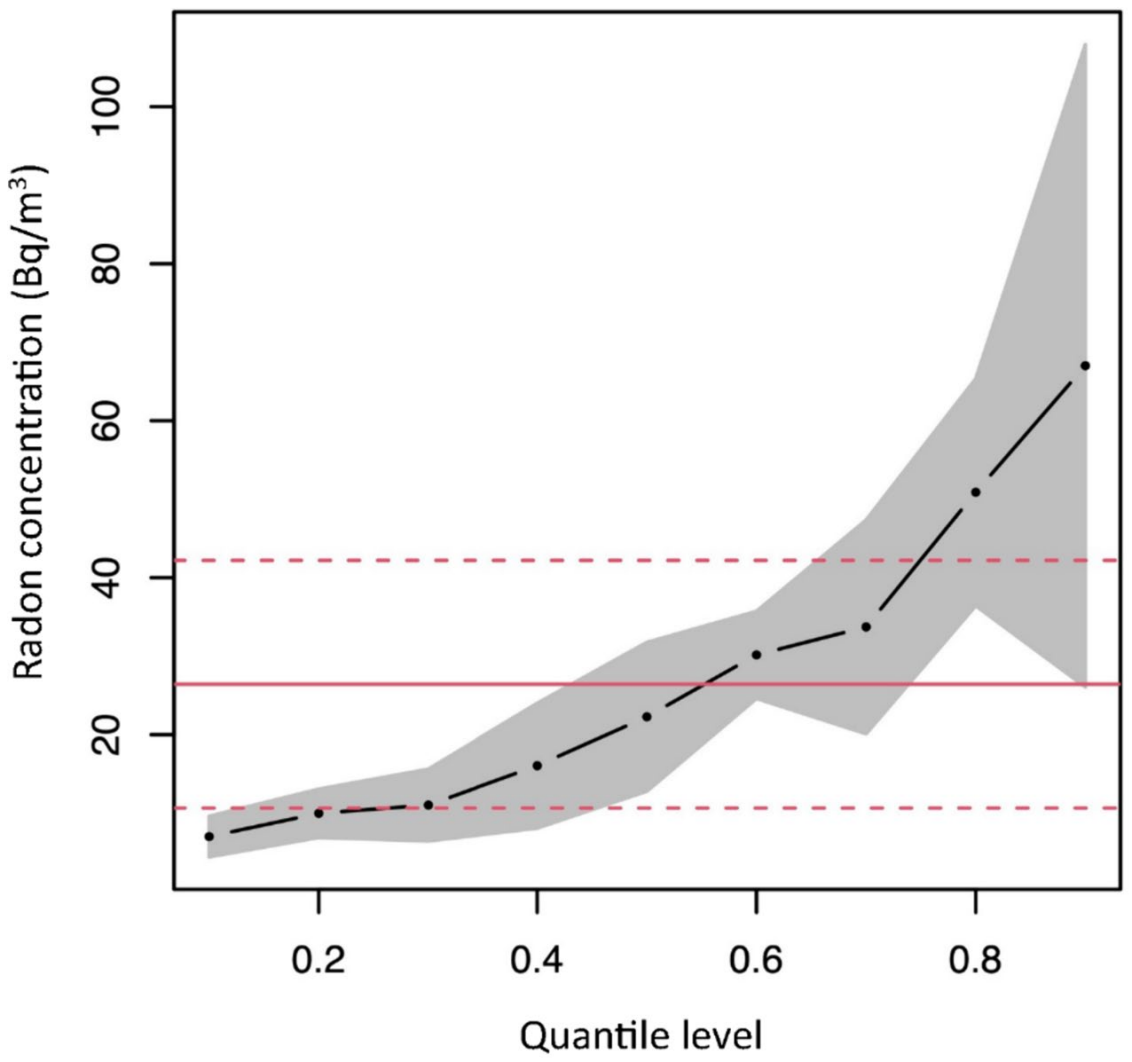
Possible parameters estimate, related to an hypothetical covariate, obtained by quantile regression.

With τ representing the quantile level of interest, reminding that a quantile of level τ (with 0< τ <1) represents the value of the Y distribution such that τ % of the data falls below it. This approach offers a regression model for each of the quantiles of interest separately. In this way, it is possible to study the influence of explanatory variables on the shape of entire Y distribution. In fact, the parameters $\beta_{\tau i}$ represent the impact of the covariate *X_i_* on the specific quantile of level τ , allowing these parameters to be different for different quantile levels (e.g., Figure 5).

The feature of different slope coefficients at different points in the distribution is particularly useful if the underlying data exhibits heteroscedasticity, that is, the quantile regression model is particularly well suited to detect and describe the heteroscedasticity and how it may act as an “effect modifier” of a predictor at different levels of the response variable (42).

The quantile regression can be applied without assuming any parametric distribution and without specifying the variance and covariance structure of the error for the response variable. The parameter estimation approach, based on the minimization of a check loss function, makes the estimates more robust than those obtained through the classical regression described by the conditional mean model (Equation 1). Quantile regression model allows to evaluate the impact of several factors simultaneously on the quantile $Q_{Y}\left(\tau \right)$ , however generally this method is not used to handle a very large number of covariates.

Starting from this original idea as presented in Koenker and Bassett (41), the quantile regression approach was further developed to make it suitable for a wide variety of data analysis settings. In particular, one extension of the quantile regression approach stems from the need to handle spatial data, such as radon concentration measurements, the difficult of which is due to the possibly highly complex spatial dependence among the various measurement sites. Moreover, the quantile regression approach was also extended to the Bayesian framework (43–45). A comprehensive review of the quantile regression class of methods, their applications, and relevant literature, is reported in the “Handbook of quantile regression” (46).

###### 3.3.2.1.1 Applications

Quantile regression can be particularly useful in the radon context, because it allows to focus on the right tail (i.e., the high percentiles) of the radon distribution, which is associated with a higher lung cancer risk.

Although a vast literature is available for quantile regression methods and many of its applications, few papers were found focusing on radon concentration (47–50), and all used methods that develop from the original quantile regression (and also incorporating spatial dependence) to evaluate the impact that predictors potentially have on high radon concentration values. In these papers the analyses are applied to indoor radon concentration datasets, in dwellings or schools. Factors included in the model are related to building characteristics (building destination of use, contact with ground, building materials, building age, dwelling floor), and in some cases also radiometric data, geological data and altitude are included.

Using a quantile regression approach, these papers generally showed that the effect of explanatory variables may change quite significantly depending on the level of indoor radon concentration (e.g., single building, direct contact with ground). In few cases no clear pattern of influence across quantiles is observed as for instances building age (50) and geological factors (49).

##### 3.3.2.2 Logistic regression

**Figure 6:**
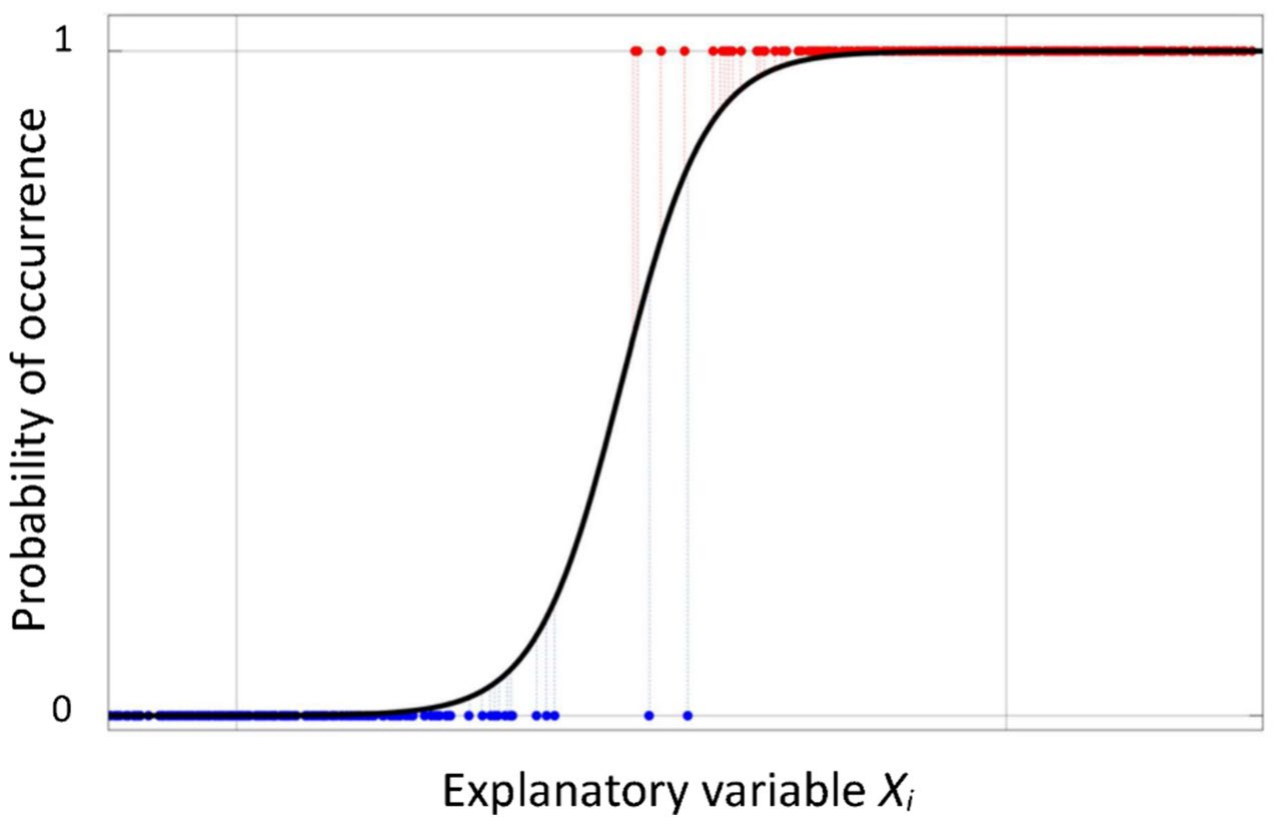
Example of logistic curve in the single-variable case.

Logistic regression is a very simple and powerful tool to develop a prediction model for a binary outcome (the dependent variable). In the present context, the binary outcome is the indoor radon concentration (IRC) above (event “1”) or below (event “0”) a certain threshold. Logistic regression is a statistical model predicting the probability *p* of an event taking place (e.g., IRC above a threshold) depending on the linear combination of one or more independent variables. Usually, when one wants to understand the relationship between one or more predictor (or explanatory) variables and a continuous response variable, it is possible to use a linear regression model. However, in our case, the response variable is categorical (high = 1/low = 0 IRC), and a classical linear regression as described in [1] is not suitable to predict the probability of the event being 1, as it is not limited in a predefined interval, while probability is (by definition, from 0 to 1). Therefore, the relationship between some predictor variables and the probability *p* to find a radon concentration higher than a fixed threshold *T* may be described through a function whose values span in the [0,1] interval (Equation 3), such as the S-shape logistic curve (Figure 6):


(3) $$p=P\left(Y>T|X_{1},,\ldots ,,X_{n}|\right)=\frac{1}{1+e^{-\left(\beta_{0}+\beta_{1}X_{1}+\ldots +\beta_{n}X_{n}\right)}}$$

Applying a proper function (i.e., logit function) to the response variable (the probability *p*), a model linearization is obtained, and it is possible to easily estimate the values of the coefficients, although their interpretation is not trivial.

Logistic regression is straightforward to use to discriminate between the many factors influencing the probability to measure high indoor radon levels, but the method requires a threshold to be set to identify what are considered high values. The threshold should be a reasonable value that considers both the data available (“high” radon concentration measurements might be a small number in a dataset) and the particular interest to use a specific threshold (because it has a regulatory or recommendatory value) (Table 4).

**Table 4:** Selected regression methods and their respective descriptions.

Method	Description
Quantile regression	Quantile regression is a statistical method for estimating functional relations between variables for all portions of a probability distribution, thus implying the possibility that there is not a single rate of change describing the relationship between a response variable and predictor variables.
Logistic regression	Logistic regression is a statistical model for a binary outcome (the dependent variable). Logistic model predicts the probability of an event taking place (in our context, indoor radon concentration above or below a certain threshold) depending on the linear combination of one or more independent variables.

###### 3.3.2.2.1 Applications

In the selected papers, the logistic model is applied using different threshold values. In Dai et al. (51), the threshold considered is 148 Bq/m^3^ (action level for remediation in the United States), while in Vukotic et al. (52) the threshold value was fixed to 200 Bq/m^3^, in line with the UE guidelines for indoor radon levels. Often different analyses are implemented using several thresholds, as done in Borgoni et al. (53), who used four different threshold values (100, 200, 300, and 400 Bq/m^3^), and in Stanley et al. (54), who used the two thresholds 100 and 500 Bq/m^3^.

All the previously discussed papers applied logistic regression with the main objective of identifying the major features influencing IRC. However logistic regression was also used to identify radon-prone areas, feeding the model not only with covariates describing building characteristics, but also with several geogenic and/or geophysical factors, including geological data and airborne geophysical parameters (29, 55, 56).

Therefore, even if logistic regression might not be entirely classified within the geostatistical methods (see next section), such an approach can also be useful to enhance predictive power of geogenic radon maps with a high level of accuracy [76.5% in (55), and 77.9% in (29)].

#### 3.3.3 Geostatistical methods

In the context of identifying buildings and areas with high radon levels, geostatistical methods have been largely explored so far. Indeed, geostatistical tools allow us to predict radon related variables based on the distribution of known observations, in areas where it has not been measured. This is possible because closer observations show higher autocorrelation than the most distant observations, which is known as the first law of geography (57): “Everything is related to everything else, but closer things are more related than distant things.” Naturally, geostatistical methods depend on georeferenced data, which is now generally used in radon surveys. They can be applied with only the data of interest or use additional georeferenced variables. Methods using many different input parameters might overlap with regression methods and machine learning methods, where for instance coordinates are only one predictor in bunch of input parameters. In this chapter, application of classical geostatistical methods such as inverse distance weight, kriging and geographically weighted regression are presented. Table 5 synthesizes some of the most used geostatistical methods in the radon context.

**Table 5:** Selected geostatistical methods and their respective descriptions.

Method	Description
Inverse Distance Weight (IDW)	Interpolation and deterministic method which relies on existing data measurements. Radius (max. Distance of influencing measurements) and power (influence pondered to distance) determine the result of interpolation.
Kriging	Interpolation and deterministic method based on existing data measurements and their statistic relationship. According to different parameters, the resulting interpolation is associated with an uncertainty map issued from the statistical modeling.
Geographically weighted regression	Multiparameter predictive tool which uses different explanatory variables, including non-stationary variables (climate, geographical coordinates), to predict an indicator. Each explanatory variable is given a coefficient, such as in a linear regression.
Local polynomial interpolation	Two-dimensional interpolation based on a polynomial function applied as a filter on a part of the total surface. Polynomial function will vary according to its location.
Global polynomial interpolation	Two-dimensional interpolation based on a polynomial function applied to the entire surface investigated.

##### 3.3.3.1 Applications

Inverse distance weight (IDW) has already and, above all, largely been applied through different studies in different contexts. Overall, authors decided to use IDW because the method has the advantage of complying well with noisy data and it might interpolate short-range variation (58–61). Radon measurements carried out in buildings might present a high spatial variability, which results in very few spatial correlations between the measurements. IDW is therefore the ideal tool to answer this kind of dataset (60). Moreover, this method presents another advantage in its ease of implementation, which makes it cost-and time-efficient. However, authors noticed some counter-performances of IDW. Since IDW allows to identify small scale variations, this method tends to miss the global trends in the overall dataset (58). Finally, the results provided by IDW are considered as valuable by the different authors (62) although it is necessary to implement IDW carefully knowing all its strengths and weaknesses.

Among all the geostatistical methods introduced in this chapter, kriging and its variations are the most implemented method to assess high radon areas (58, 59, 61, 63–69). Most of authors note the difficulty to include all related elements and carry out the different steps of the methodology, although some software allows to simplify its application, such as ArcGIS (65, 66). Nevertheless, this method presented really good results: Sabbarese et al. (68) concluded that kriging allowed them to identify radon prone areas even the ones where very few data was recorded. Similarly, a geogenic radon potential map, highlighting radon-prone areas was created using kriging and co-kriging (67). Moreover, it has been highlighted that kriging is the best linear unbiased spatial predictor (58, 61). It has been noticed that an application of kriging algorithm allows to avoid smoothing effects (63). Beyond the difficulty to implement this method, it has been shown that the calculated weights not only depend on the distance but also on the direction and orientation of the closest data in scarce data area. Cafaro et al. (65) underlined a strong need to deeply understand radon’s underlying layers, such as geology, and to have a homogeneous spatial repartition of radon data among the studied spatial extent. Indeed, unreliable measurements might be produced by non-stationary effects and the lack of correlation between geology and indoor radon levels, especially in karstic areas (65). Akkala et al. (58) also underlined non-stationarity issues in real-world datasets. Kriging may be declined in different models, such as simple, universal, conditional or disjunctive kriging (70). The latter model revealed to be very effective to identify radon prone areas using only indoor radon measurements (69). Finally, Bachirou et al. (59) compared the performances obtained with ordinary kriging and IDW. No significant differences were observed in the prediction errors of the two techniques adopted, applied in a similar context and with an identical dataset (59).

Then, geographically weighted regression (GWR) has been used several times to identify radon-prone areas. This method has been applied several times in Italy (71–73). GWR presented the advantage of localizing small-scale variations, such as IDW, but also produces a map of the local coefficient (73). Ciotoli et al. (71) underlined the accurateness of the model, which is commonly used, and the fact that only influential explanatory variables are needed to run the model. Finally, De Novellis et al. (72) used GWR because it allowed to estimate the indoor radon concentration by using some local environmental properties, e.g., the geogenic radon potential of the underlying soil. Although GWR presents good results, revealed by the authors, this method is very sensitive to the representativeness of the sampled data (73).

Other geostatistical methods were investigated and compared in two articles (58, 61). Local and global polynomial interpolations were applied, and their results are compared to other methods, such as kriging, IDW and machine learning methods. LPI presented the advantages of being easy to implement, to comply well with noisy data, and to interpolate local variations, while the estimation errors increased exponentially with increasing complexity in the training dataset. GPI requires less data to interpolate and was computationally less intensive than LPI. However, GPI was not suitable for extrapolation. Compared to IDW and kriging, these two methods presented lower performance scores (normalized mean square error) than kriging ones, while the latter seemed to be similar as IDW accuracy (58, 61).

Finally, some authors used GIS (Geographic Information System) software to implement different and less common methods, such as Rasters Factor Rating Method (74), PCA and correlation analysis (75), Bayesian estimation of the percentage of houses exceeding a given action level (76).

#### 3.3.4 Machine learning methods

Machine learning is a collective term for applications using statistical models to analyze, make predictions and draw inferences from data, without following full instructions. Machine learning methods can be divided into three main categories: supervised learning, unsupervised learning and reinforcement learning. In the field of radon usually supervised and, in a few cases, unsupervised machine learning methods are used. Supervised learning uses labelled input data to generate models to predict unseen or future data. Machine learning methods have been shown to be very successful in many applications and are already used in predicting radon levels, as summarized and analyzed also in recent studies (77, 78).

Supervised learning with a numerical target value is a regression task which is already described in the quantile regression section (section 3.3.2.1.). Unsupervised learning analyses data and tries to find underlying patterns and structure in the data. As examples Table 6 presents different selected methods applied in the framework of radon prediction.

**Table 6:** Selected machine learning methods and their respective descriptions.

Method	Description
Random forest	Assemble of decision trees, applicable for regression and classification task. Can handle categorical and/or numerical predictors without further modifications.
Support vector machines	Linear boundaries as lines, planes or hyperplanes are used either to separate binary classes or used as prediction in a regression task. It can be extended to non-linear models using the kernel trick, where predictors are transformed to higher dimension, where non-linear relations become linear separable.
Feed forward neural network	An input is passed through connected neurons to an output. The error at the output layer, where a loss function measures the performance of the model predictions, is used to backpropagated to adjust the weights and biases of the neurons.

Supervised learning predictive models are built using labelled training data. Model performance is evaluated on test data using metrics (e.g., loss functions) to calculate differences between the prediction and actual value (e.g., accuracy, confusion matrix, mean squared error). The prediction can be a categorical or numerical value and, based on the target value and the aim of the prediction, a suitable metric is used. A great variety of different supervised learning methods for various applications exist.

In the field of radon, various machine learning methods have been used already, especially for the prediction of indoor radon concentrations. The reason for using data-driven machine learning models is that a valid physical transport model from the soil to actual indoor radon concentrations has not been found until now. The process depends on different factors, that also might be independent from each other such as the geogenic radon potential, the type and the technical standard of the building and the usage of the building. Also, in recent years many indoor radon concentration surveys have been carried out, which is a solid data basis for machine learning techniques.

##### 3.3.4.1 Applications

Overall, 18 publications were selected and in the following their ability to predict high radon levels is summarized.

The selected papers showed a great variety of machine learning models. Some of the publications test different models or use several ensembled models. The most used supervised machine learning methods are artificial neural networks (58, 61, 78–81), followed by random forest (79, 82–84). Other models are only used in one publication: support vector machine (84), convolutional neural network (85), LSTM (86), k-nearest neighbor (87), mixed effect regression model (88), extreme learning machine (86), random vector functional (86), multivariate adaptive regression splines (84), boosted generalized additive and linear model (80), XGBoost (78, 79), automatic linear modeling (79), and group method of data handling (89). In four publications, unsupervised machine learning methods or semi-supervised machine learning methods, Bayesian cluster detection (50), k-medoids clustering (83), k-mean clustering (90) and Bayesian profile regression were used. None of these methods are specifically designed to focus the prediction on high radon levels. In the following the overall workflow of the selected publication is analyzed, focusing on modifications of the target variable and the used train/test split strategy.

Indoor radon concentrations, their aggregates or transformations, were the target value in 15 publications. In two publications, the geogenic radon potential was used as target variable. In three publications, the actual IRC value was used as target value which keeps the focus on high radon levels, because single high values have a greater potential for prediction errors, as also discussed in the result section. In one publication the indoor radon concentrations were transformed in a classification task with three classes (low, high medium). In two other publications the probability exceeding a reference value is used as target value. Doing so, the former regression task is also transformed to a classification task. Both are valid strategies to predict high radon levels. In the remaining publications, log-transformed indoor radon concentrations or aggregates like the geometric mean are used for prediction. Although these models are also capable of predicting areas with high radon levels in general, they are not specially designed to predict high radon levels, but rather focus on predicting average or mean values. Still a well-designed machine learning workflow can be a very powerful tool for predicting radon levels and high radon levels. Overall, the selected papers usually do not include detailed sections of the modification of the target value (e.g., the log-transformation, aggregation) or the reason why a certain modification was used, with some exceptions discussed in more detail in the following.

Another sensitive topic when applying predictive models is the model evaluation on training and test data. In 14 publications the data splitting in training and test data is documented. In 12 of these, the data split was performed randomly without further consideration. In two publications the data split was performed on spatial criteria to avoid spatial autocorrelation of the target variable. When comparing the model performance, it is essential to consider if it was evaluated on the training, validation, or test data or if these account for the spatial autocorrelation of the target value. Models without a clear data split workflow might show high model performance, but will fail to generalize and might perform poorly on new or unseen data. Keeping these general remarks in mind, we will now give short descriptions of selected publications with different workflows, that can serve as profound examples and baseline of a machine learning workflow.

In Petermann et al. (84) three different machine learning methods (random forest – RF, support vector machines – SVM, multivariate adaptive regression splines – MARS) are tested to predict the geogenic radon potential in Germany with 36 possible predictors. The autocorrelation of the target value is described and analyzed, and a solution outlined how to deal with this autocorrelation by splitting training and test data in geographical blocks. Leave-one-out cross validation was used to explore which predictors are useful and to tune the hyperparameters of the three models. Feature importance for the predictors was calculated and an in-depth analysis of their impact on the results was carried out. Multiple metrics for performance evaluation are given and the distribution of the prediction is discussed, stating that the predictions tend to overestimate small values and underestimate high values. Performances metrics for MARS, RF and SVM were, respectively, 55.2, 52.8 and 54.4 for RMSE, 0.16, 0.22, 0.22 for *R*^2^, and 25.6, 23.7 and 22.2 for MAE, thus denoting a slightly higher predicting power of Random Forest (RF). The publication is the only one of the selected publications that does not predict indoor radon concentrations but covers so many aspects of a profound machine learning workflow that it can serve as a very good starting point for predicting a spatial autocorrelated target value. In the publication of Rezaie et al. (86), three different machine learning methods (long short-term memory – LSTM, extreme learning machine – ELM, random vector functional link – RVFL) were tested to predict the non-transformed indoor radon concentration. The training and test split were done randomly which does not take autocorrelation into account. The models are complex models in the field of neural networks. The introduction and the discussion of the results are in-depth analysis also using different performance metrics. LSTM, ELM, and RVFL models performance were similar, as depicted by their respective AUC-ROC score of 0.81, 0.83, and 0.82.

In Wu et al. (78) two machine learning methods (Neural-network and XGBoost) are used to predict radon levels. The usually applied regression task is transformed to a classification task with three radon classes based on the Swedish radon legislative: 0 to 200 Bq/m^3^, 200 to 400 Bq/m^3^, and greater 400 Bq/m^3^. Among the selected publications this is a unique approach and relevant for also predicting high radon levels, As also stated in the result section machine learning models predicting classes, might be more robust compared to regression workflows, and therefore might also predict high values more accurately. The train and test split and the performance metric is clearly described and documented, XGboost achieved better results than the neural network: macro-F1 score were, respectively, ranging between 0.93–0.96, and 0.64–0.74 for XGboost and neural network. Interestingly the accuracy of the developed model was highest for the low and high classes. The middle class showed the highest errors among the three classes. The study shows that the transformation from a regression to a classification task could lead to more robust predictions for high radon values. The downside is that by using classes as a target variable, information about the actual numerical radon level gets lost. A comparison with a regression model using the same data and workflow would be interesting but was not applied.

In two selected publications unsupervised machine learning techniques are also applied. In Kropat et al. (83) lithological units based on their IRC distribution are clustered in classes. Six classes are later used for the prediction of IRC among other predictors to predict indoor radon concentrations with regression trees. When using such a lithological classification based on the target value, information flows from the target value to the predictor space. This could lead to models that might not generalize well and overestimate the actual importance of the lithological classes as predictors. But still, the clustering of lithological units alone can be a very interesting method, when searching for high-concentration areas or by producing an actual radon map. In Sarra et al. (50) supervised and unsupervised learning techniques were used together. In this publication, a quantile regression model was built on building characteristics to predict indoor radon concentrations in standardized homes. These standardized indoor radon concentrations best reflect the geogenic radon potential. In a second step the distributions of the standardized radon concentrations are used to cluster lithological units into areas of radon hazard.

### 3.4 Assessment of performance metrics

With regards to the various applications identified within the literature review conducted using the PRISMA method, we provided a synthesis table of performance metrics used to assess performances of the different methods. Table 7 lists and highlights the connections between the various methods, if any.

**Table 7:** Synthesis of performance metrics with their relative description and relationships.

Category	Performance metric	Description	Relationships with other metrics
Classification	ROC-AUC	Measures the ratio between the true positive rate and false positive rate, represented by the area under the ROC curve.	AUC provides a single metric to compare different models’ ability to identify different classes.
	Confusion Matrix	Table listing true positives, true negatives, false positives, and false negatives, which help at describing classification performance.	Basis for calculating precision, recall, F1-score, and accuracy.
Regression	Mean Absolute Error (MAE)	Average of the absolute differences between predicted and observed values.	MAE is less sensitive to outliers compared to MSE and RMSE. Often compared with these metrics to assess error distribution.
	Mean Squared Error (MSE)	Average of the squared differences between predicted and observed values, more sensitive to outliers.	Squaring amplifies the impact of larger errors (outliers), leading to more conservative models compared to MAE.
	Root Mean Squared Error (RMSE)	Square root of MSE, having the same units as the original data.	Directly related to MSE. RMSE is easy to interpret due to the same unit as the original dataset.
	*R*-squared (*R*^2^)	Part of variance in the dependent variable that is predictable from the independent variables. *R*^2^ ranges from 0 to 1 and can be expressed as a percentage	Often compared with Adjusted R-squared to assess the impact of adding more predictors.
	Adjusted *R*-squared (Adj. *R*^2^)	*R*-squared adjusted to the number of predictors included in the model. Score decreases with the addition of non-significant predictors.	Adjusts *R*-squared to prevent overfitting by adding too many predictors.
Model selection	Akaike information criterion (AIC)	Measure of the relative quality of statistical models, by balancing fit and complexity of the model.	Often compared with BIC. Both metrics penalize model complexity, but AIC is less strict.
	Bayesian Information Criterion (BIC)	Similar to AIC but with a stronger penalty for models with more parameters.	Stricter than AIC, and often preferred when overfitting is a concern.

## 4 Discussion

As demonstrated by the results, various methods exist for identifying radon-prone areas and buildings, ranging in complexity and implementation. These methods encompass a spectrum of approaches, from basic statistical methods to AI methods. Based on our findings, the present study led to the production of Table 8, delineating the strengths and weaknesses inherent in each method’s category. This evaluation sheds light on the efficacy and limitations of each approach, aiding in the discernment of optimal methodologies for high radon levels prediction. Moreover, specific circumstances under which each method proves to be the most suitable are highlighted, thus facilitating an informed decision-making regarding the selection and deployment of radon evaluation strategies in diverse environmental and geographical contexts. Table 8 provides guidance for stakeholders involved in radon risk assessment and management efforts.

**Table 8:** Strengths and weaknesses of the different classes of method identified.

Category	Strengths	Weaknesses	Recommendations
Descriptive statistics	Provide good results while being easy to implement.	Some analyses are subject to interpretation.	Strong knowledge of statistics and the data is required. Useful to guide on more specific and appropriate analyses.
Regression	Fairly well-known and widespread method; produces results that are fairly easy to interpret and that are fairly familiar to many people	It works better when the number of covariates is not very large. Certain prerequisites/assumptions must be verified for applicability. To perform a logistic regression, a specific reference level should be fixed.	Strong knowledge of the dataset and of the relationships between variables is required. Certain. Assumptions on which the model is based should be checked.
Geostatistical tools	Powerful and adapted methods to predict radon levels where it has not been measured.	It is sometimes time-consuming and difficult to apply. Many parameters must be included.	Spatial behavior of the predicted variables and predictors must be known.
Machine learning tools	Various methods and strategies to deal with different research aims and scopes, the most powerful predictive models available.	The implementation and learning can be difficult and computationally expensive. Usually, black boxes and additional analysis for interpretation is needed.	Clear workflow from data handling to performance evaluation, as modifications of the target variable or the train/test split strategy.

Recommendations of use are provided by the authors, based on the literature review.

Overall, the methods investigated in our research consistently yielded results aligning with researchers’ expectations. Basic statistical methods demonstrated robust performance alongside ease of application, guaranteeing a comprehensible analysis. Quantile and logistic regression methods emerged as effective tools for forecasting elevated indoor radon levels, offering both simplicity in implementation and clarity in interpretation. Meanwhile, geostatistical methods, with their spatial component, emerged as efficient in predicting indoor radon concentrations in unmeasured areas, thereby facilitating the identification of radon-prone regions. However, researchers must take care in methodological application and parameterization to ensure accurate and reliable results. Finally, machine learning methods examined in the analyzed papers are generally not tailored specifically for predicting high radon values. Still, the methods applied can be used to specially target high radon levels and even more with the modifications discussed in section 3.2., as adoption of the loss function or keeping the target value on linear scale. An impact assessment of the inclusion of different modifications within ML methods to specifically predict high radon levels must be more explored.

These findings underscore the significance of methodological choice and careful consideration in the pursuit of effective high radon risk assessment. More generally, the analyzed publications used diverse data sources, manipulations, models, and performance metrics, making it challenging to compare them and draw definitive conclusions about the most effective workflows for predicting high radon areas and buildings. Consequently, we propose further research to determine the best-practice among the methods investigated tailored for predicting elevated radon levels and to determine the optimal strategy aimed at identifying as many dwellings as possible with high radon concentrations.

While our literature review synthesizes key insights regarding the identification of radon-prone areas and buildings, our study still presents some limitations. The application of methods across varied datasets, transformed and homogenized with various prior manipulations, models, and contextual settings, introduces a significant challenge to direct comparison. Moreover, the absence of standardized metrics for method performance evaluation complicates the assessment process, hindering the ability to benchmark the different approaches. Additionally, the interpretation of strengths and weaknesses within literature often remains subjective, influenced by authors’ individual perspectives and experience. These limitations underscore the need for further refinement and standardization in methodologies to enhance the robustness and comparability of research findings in this critical field of study.

## 5 Conclusion

This paper aims to provide researchers with a systematic literature review of the various methods employed to identify radon-prone areas and buildings. Through our review, based on the PRISMA methods, we identified different methodologies that can be categorized into four main classes: descriptive statistics, regression methods, geostatistics, and ML methods. While these categories were established by the authors, they are not always distinct, as certain methods may fall into multiple classes. However, this classification has enabled to highlight diverse application contexts alongside their corresponding results. By examining the available techniques within each category, this paper offers insights into the effectiveness and applicability of different approaches for addressing the challenge of the identification of radon prone areas and building, i.e., areas and buildings with high radon levels.

The investigated methods primarily focus on identifying the predictors of elevated concentrations or delineating the characteristics of clusters within buildings and areas with heightened levels of radon. The methods tackled in this study serve dual purposes: identifying radon prone buildings and radon prone areas. Nevertheless, machine learning and geostatistical approaches are predominantly deployed to identify areas with elevated radon levels, often due to the availability of georeferenced data. Conversely, quantile and logistic regression methods are more frequently utilized to identify predictors of elevated radon concentrations within buildings. However, in some recent studies, these regression methods’ original iterations have been expanded to accommodate the spatial correlation inherent in radon concentration measurements.

After evaluating the diverse papers and their application of various methods, several questions emerged for consideration: at firstly, there is a query regarding whether it is advantageous to employ specific methodologies that focus on high radon concentrations, as opposed to more conventional methods targeting average (geometric or arithmetic) levels. Notably, one method discussed in this review, quantile regression, demonstrated the potential for certain explanatory variables to exhibit varying impacts depending on the indoor radon concentration targeted. Secondly, a concern arises regarding the extent to which the outcomes of the applied methods rely on the characteristics of the dataset under analysis. Factors such as the age of the datasets or the geographic area from which the data are gathered may significantly influence the results, particularly concerning the identification of factors impacting high indoor radon levels.

For instance, when applying logistic regression to study different datasets, containing very different sets of predictors, the results as expected are variegated while generally in agreement; however, in some cases the use of different dataset may lead to apparent contradictions. For instance, in Stanley et al. (54) old buildings were found to have lower probabilities of being associated with high IRC while in Vukotic et al. (52) the findings were the opposite. Clearly the concept of “old” has two very different meanings in Europe and in North America and the types of buildings considered were not easily comparable.

Our analysis revealed the challenge of comparing the different methods due to the multitude of datasets, data manipulations, models, and performance metrics involved. Indeed, it is quite difficult to compare different analyses on different datasets, applying different investigation strategies and different statistical approaches, since (1) the performance of a method strongly depends on the dataset under examination and even on the outcome variable (e.g., radon concentration on a continuous scale, on a binary scale, on a log-scale), (2) the performance metrics/indicators used are different depending on the model applied. Results exhibit a high degree of dependency on local factors and the characteristics of the dataset being analyzed. As a result, it becomes evident that there is no universally optimal method applicable across all scenarios. Rather, the selection of the optimal method is contingent upon the specific attributes of the available dataset and the practical feasibility of applying a particular methodology. This underscores the importance of carefully considering the nuances of each situation when choosing an appropriate approach for identifying and addressing effectively high indoor radon concentrations.

This paper represents the initial step to review and evaluate the diversity of methods used in identifying radon-prone areas and buildings. Serving as a foundation for future work, this paper lays the groundwork for applying methodologies, as outlined herein, to available datasets. The challenge is to come to a deeper comprehension of how different methods perform when applied to the same dataset, thereby enhancing our ability to effectively address a robust comparison. Through this paper, we hope to offer valuable insights as a basis to guide future efforts in the identification of radon prone areas and buildings, and ultimately, reduce the population exposure.

## Data Availability

The original contributions presented in the study are included in the article/supplementary material, further inquiries can be directed to the corresponding author.
